# A Disorder-Induced Domino-Like Destabilization Mechanism Governs the Folding and Functional Dynamics of the Repeat Protein IκBα

**DOI:** 10.1371/journal.pcbi.1003403

**Published:** 2013-12-19

**Authors:** Srinivasan Sivanandan, Athi N. Naganathan

**Affiliations:** 1 Department of Biotechnology, Indian Institute of Technology Kharagpur, Kharagpur, India; 2 Department of Biotechnology, Indian Institute of Technology Madras, Chennai, India; Fudan University, China

## Abstract

The stability of the repeat protein IκBα, a transcriptional inhibitor in mammalian cells, is critical in the functioning of the NF-κB signaling module implicated in an array of cellular processes, including cell growth, disease, immunity and apoptosis. Structurally, IκBα is complex, with both ordered and disordered regions, thus posing a challenge to the available computational protocols to model its conformational behavior. Here, we introduce a simple procedure to model disorder in systems that undergo binding-induced folding that involves modulation of the contact map guided by equilibrium experimental observables in combination with an Ising-like Wako-Saitô-Muñoz-Eaton model. This one-step procedure alone is able to reproduce a variety of experimental observables, including ensemble thermodynamics (scanning calorimetry, pre-transitions, *m*-values) and kinetics (roll-over in chevron plot, intermediates and their identity), and is consistent with hydrogen-deuterium exchange measurements. We further capture the intricate distance-dynamics between the domains as measured by single-molecule FRET by combining the model predictions with simple polymer physics arguments. Our results reveal a unique mechanism at work in IκBα folding, wherein disorder in one domain initiates a domino-like effect partially destabilizing neighboring domains, thus highlighting the effect of symmetry-breaking at the level of primary sequences. The offshoot is a multi-state and a dynamic conformational landscape that is populated by increasingly partially folded ensembles upon destabilization. Our results provide, in a straightforward fashion, a rationale to the promiscuous binding and short intracellular half-life of IκBα evolutionarily engineered into it through repeats with variable stabilities and expand the functional repertoire of disordered regions in proteins.

## Author Summary

It is well recognized that unstructured or disordered proteins play a vital role in the cell. How does this disorder translate into function, and what effect does it have when linked to ordered regions? We attempt to answer this question by computationally characterizing the repeat protein IκBα, a central player in the NF-κB signaling module that possesses both structured and disordered domains. Upon constraining a structure-based statistical mechanical model with equilibrium experiments, we are able to successfully predict both the ensemble kinetic and single-molecule behaviors. Functionally, we unearth a unique mechanism in which the effect of disorder propagates, even into ordered regions, in a domino-like fashion, thus rendering the entire structure highly flexible. In other words, the evolutionarily designed disorder in IκBα places it on a functional precipice that can be either recruited for binding to transduce external stimuli or just be degraded to shut down the inhibitory effect, reconciling both functional and folding behaviors in a single framework.

## Introduction

NF-κB, a mammalian multi-domain transcription factor family, regulates immune responses and the expression of several genes associated with cell growth and diseases through a tightly regulated signaling pathway [1]. In its inactive state, NF-κB dimer is tightly bound to IκBα thus localizing the complex in cytoplasm and inhibiting transcription. Upon the presence of appropriate signals, IκBα is degraded releasing NF-κB that enters nucleus and initiates the transcription of hundreds of essential genes [1], [2]. Apart from its fundamental role in inhibiting transcription, IκBα is also known to bind at least 10 different proteins with the affinities for some complexes varying by over 3 orders of magnitude [3], [4]. From a biological regulatory standpoint, IκBα thus plays a critical role in the proper functioning of the cellular machinery and its response to external stimuli as it is also implicated in Hodgkin's lymphoma [5].

**Figure 1:**
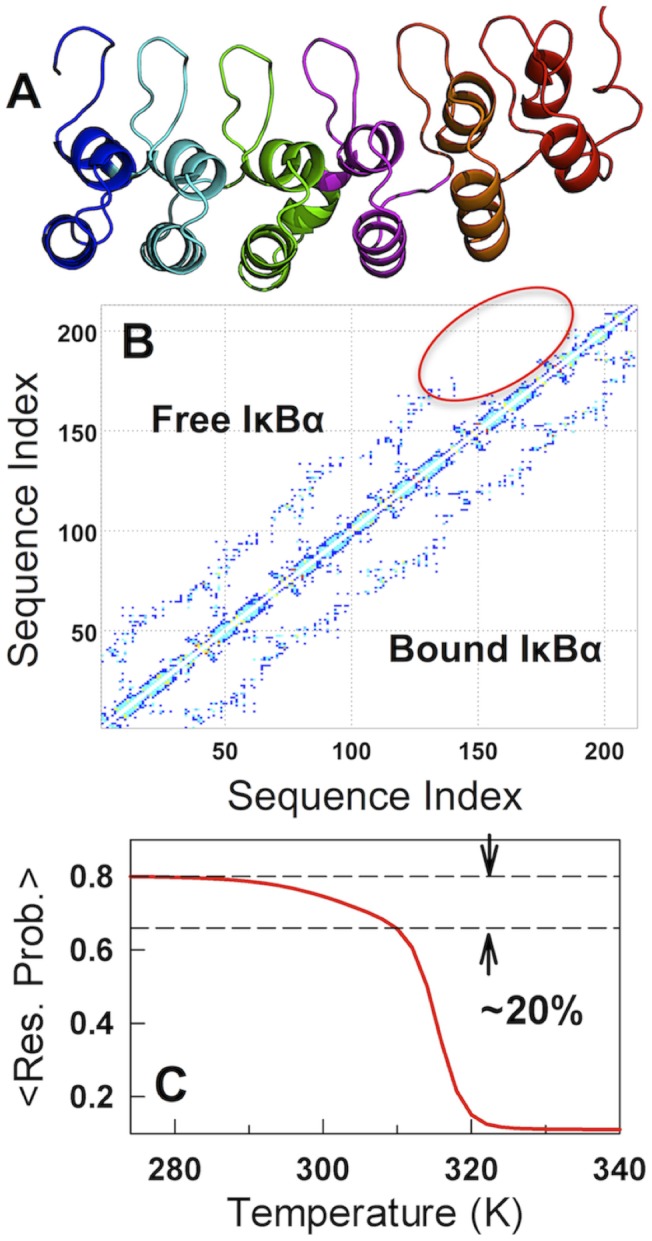
Modeling disorder in IκBα and thermodynamics. (A) Crystal structure of the bound-IκBα. (B) Contact-map of bound- and modeled free-IκBα. It can be seen that the repeats 5 and 6 in the modeled free-IκBα (circled region) are dominated by local contacts. (C) The predicted unfolding curve for the free-IκBα with a prominent pre-transition that accounts for ∼20% of the total unfolding amplitude.

Structurally, IκBα is composed of the N-terminal signal response region that targets the protein for ubiquitination, the central 6 ankyrin repeat (AR) domain [6], and the C-terminal disordered PEST region [7]. True to its promiscuous binding and central role in signaling, the domain composed of the 6 ARs (from hereon referred to as IκBα; Figure 1A) displays a complicated solution behavior and resists crystallization in its free-state (i.e. in the absence of binding partners) unlike its counterpart in Bcl-3 [8] and short half-life within cells (<10 minutes) [9]. An array of ensemble biophysical measurements on IκBα from the group of Komives and co-workers indicate a malleable conformational behavior with large ANS binding (molten-globule like character), low thermodynamic stability (melting temperature ∼315 K compared to the physiological growth temperature of 310 K) [10], repeat-specific hydrogen/deuterium exchange pattern and hence different stabilities with repeats 5 and 6 significantly disordered [10]–[12], and significant roll-over in the denaturant dependence of relaxation rates indicative of multiple intermediate states during (un)folding [13]. Recently, ensemble experiments have also been buttressed by some elegant single molecule Förster Resonance Energy Transfer (smFRET) experiments on IκBα with the donor/acceptor placed at different repeats (AR 1–4, 2–5, 2–6 and 3–6) thus monitoring the overall distances between the different repeats that can be used as a proxy for the degree of unfolding. These experiments reveal that the distance between the repeats evolve in an intricate manner with the number of peaks, their mean FRET value and broadness, dependent on not only the identity of the repeats but also the temperature [14], [15].

Though the experiments are comprehensive and probe a variety of conformational features, there are several questions that remain unanswered that includes the reason for its low stability, the unique behavior of the individual repeats, structural properties of the intermediates, the magnitude of the barriers separating them, the possible conformational states that are populated in smFRET experiments, and in essence the nature of the entire folding free energy landscape. Identifying the key conformational states are necessary as they directly impinge on the unique folding behavior of IκBα and the possible reasons for its promiscuous binding role. A related issue is the structure of IκBα that is characterized by both ordered and disordered regions – the so-called supertertiary structure [4] – that goes beyond the conventional modeling procedures developed for purely ordered or intrinsically disordered proteins. In other words, any model dealing with IκBα has to adequately capture both these distinct behaviors while simultaneously reproducing experiments (including smFRET) to make predictions of value.

Here we address these issues by employing the structure-based Ising-like statistical mechanical Wako-Saitô-Muñoz-Eaton model [16]–[18] (WSME) with electrostatics [19] and solvation terms [19], [20] that has a higher level of resolution and predictive capability [19], [21] compared to other Ising-like studies on repeat proteins [22]–[27]. We predict in a semi-quantitative fashion several details of the landscape and show how the lack of structure in a single repeat can have a dramatic effect on the resulting landscape with implications on extending the functional repertoire of disordered regions.

## Methods

The Wako-Saitô-Muñoz-Eaton (WSME) model is a structure-based Ising-like statistical mechanical model [16]–[18] that is consistent with the minimal frustration principle of the energy landscape theory [28], i.e. native contacts are assumed to determine the folding mechanism. Briefly, the basic surmise is that the conformational space of every residue can be represented as binary variables 0 and 1, for unfolded and folded subspace, respectively, with multiple nucleation sites distributed along the chain. The large cost involved in fixing an unfolded residue in a native-like conformation is taken into account by the entropic parameter Δ*S_conf_*. In the exact solution formulation of the WSME model, the contribution to the partition function from a very large ensemble of *2^N^* microstates is taken into consideration. The effective stabilization free-energy of every microstate is represented as a sum of van der Waals interaction energy (*ξ*), electrostatics (based on the Debye-Hückel model) and solvation free-energy (that depends on the heat capacity change Δ*C_p_*) (equations S.2–S.6 in the supporting text S1). A detailed description of the method and energetic terms can be found in two recent works [19], [21] and also in the supporting information file (supporting text S1). The global partition function can be calculated from a transfer-matrix formalism (equations S.7–S.8), which enables the calculation of both free-energy as a function of number or ordered residues (by accumulating partial partition functions) and the global probability of finding a residue folded (equation S.9).

It is important to note that though the WSME model is similar to the analytical model of repeat proteins developed by Ferreiro et al. [24], it is different in several aspects. Firstly, the WSME model has not only been employed for repeat proteins but also globular proteins [18], to predict mechanisms of folding [20], [29], [30], to engineer stabilities through mutations [21] and even in the analysis of pulling experiments [31], [32] and in characterizing the effects of confinement [33]. Secondly, the WSME model in the current version includes contributions from *2^N^* microstates (where *N* is the protein length) with detailed mean-field energetics (van der Waals, electrostatics and solvation) while the model of Ferreiro et al. [24] does not make these distinctions and employs a much smaller ensemble of just *2^R^* microstates where *R* is the number of repeats. However, some of the predictions are quite similar from these models despite these differences and we discuss them below.

In the current work, van der Waals interaction partners are identified with a 6 Å cut-off excluding the nearest neighbors. The effective dielectric constant (*ε_eff_*) is fixed to 29 that has been successful in reproducing the equilibrium and dynamic behavior of four different homologous families and also the thermodynamic effect of 138 single point mutations involving charged residues from 16 different proteins [19], [21]. Charges on IκBα were assigned according to the experimental pH 7.0 protonation state and the ionic strength value was fixed to 0.05 M [10]. The partition function and the overall probability of finding a residue folded was calculated using the methodology of Wako and Saitô [16], [17]. The final parameters are: Δ*S_conf_*  = −18.1 J mol^−1^ K^−1^ per residue (at a reference temperature of 385 K [34]), *ξ* = −70.1 J mol^−1^ per native contact and 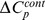 = −0.358 J mol^−1^ K^−1^ per native contact [19]. Chemical denaturation effects are introduced by a phenomenological constant (*m^cont^*) that decreases the stabilization free-energy linearly with denaturant concentration (equation S.10).

The Single Sequence free-energy landscapes are calculated by algorithmically enumerating the statistical weights of single stretches of folded residues employing the same parameters as above. Though this dramatically reduces the complexity of the folding landscape, it provides a simple minimalistic way to visualize it. Moreover, such a representation has already been successful in identifying experimentally consistent intermediates in the folding of bovine lactalbumin [19]. Since this landscape is characterized by single-stretches of folded-like protein their structure can be directly obtained by editing the PDB file [29] that enables one to calculate experimental observables (number of hydrogen bonds, contacts, contact-order, secondary structure) for a zeroth-order approximation of the possible signal along the one-dimensional free-energy profile. A similar methodology was employed to calculate distances between the labeled fluorophores from the crystal structure (PDB id: 1NFI) for the structured regions. Since the number of unstructured residues in the disordered regions are known it is possible to approximate the distance from the freely-jointed chain model [35] that is commonly employed to interpret single-molecule FRET measurements [36]:
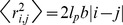
where the left hand side corresponds to the mean squared distance between residues *i* and *j*, *l_p_* is the persistence length (fixed to 4 Å) and *b* is the bond-length (fixed to 3.8 Å). The energy transfer efficiency (*E*) is then calculated from
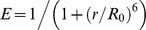
where *R_0_* is the Förster radius.

## Results

Equilibrium experiments on free (unbound) IκBα reveal that ∼20% of the total transition amplitude can be accounted for by a steep pre-transition that has been termed as ‘on-cooperative’ [37]. This can be seen as a subtle version of a related observation in an archaeal AR protein in which an equilibrium intermediate is populated [38] or a variant of the near-continuous transitions observed in alpha-helical downhill-folding proteins [29], [39], [40]. Evidence from hydrogen-exchange measurements, mutational studies and tryptophan fluorescence suggest that the pre-transition originates from the weak structure of AR domains 5 and 6 [37]. Accordingly, we systematically eliminated interactions between those repeats and the rest of the protein (bound IκBα; Figure 1B) until it was possible to reproduce the magnitude of the pre-transition observed in equilibrium experiments (Figure 1C) while at the same time maintaining agreement with differential scanning calorimetry (DSC) measurements [10] that enable a precise estimate of the sharpness or co-operativity of the global unfolding transition (Figure S1A). The resulting contact-map (Figure 1B) lacks long-range interactions beyond 15 residues in the ARs 5 and 6 (i.e. dominated entirely by local interactions) and thus serves as a physical template that accounts for the presence of order and disorder in the same structure. This is further validated by the fact that we are able to capture the *m*-value for chemical denaturation upon characterizing just the thermal denaturation data (Figure S1). Global thermodynamic analysis of IκBα folding therefore points to a two-state-like picture with sharp transitions in equilibrium experiments albeit with a significant pre-transition.

**Figure 2:**
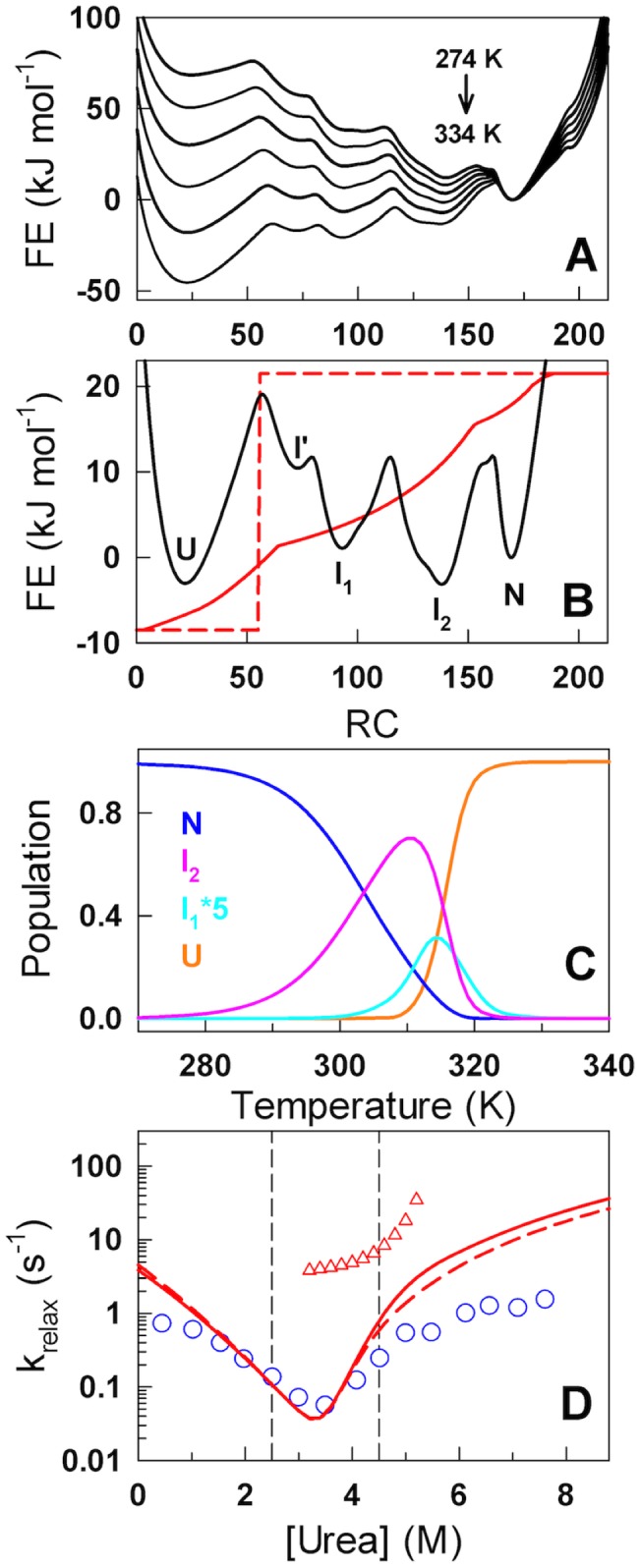
Intermediates and chevron roll-overs. (A) The predicted 1D free-energy profiles as a function of the number of structured residues (RC – reaction coordinate). (B) A closer look at the free energy profile close to the denaturation midpoint indicating the presence of two major intermediates (I_1_ and I_2_), a minor intermediate (I′) apart from the end states (U and N). The continuous red curve is the tryptophan signal calculated from the 2D structural ensemble while the dashed red curve is the assumed tryptophan signal switch. (C) Population of the major macrostates as a function of temperature. (D) Relaxation rates predicted by the model (continuous and dashed lines) compared with experiments (circles). The predicted rates were matched with the experimental midpoint relaxation rates assuming a single uniform diffusion coefficient of (7e6/*N*) n^2^ s^−1^ where *n* is the reaction coordinate value and *N* is the protein length. Triangles correspond to the faster phase observed in simulations.

However, kinetic experiments on IκBα and its variants indicate that at least 4 thermodynamic macrostates – two intermediates and two end-states (native and unfolded) - are necessary to explain the dependence of relaxation rates on denaturant, *i.e.* the chevron plot, though the magnitude of barriers and the identity of the intermediates are unknown [13]. To reconcile these two observations we calculated the one-dimensional (1D) free-energy profiles as a function of temperature with the number of structured residues as an order parameter (Figure 2A). Under folding conditions, the lowest free energy is observed at a value of ∼170 structured residues that corresponds to structured ARs 1–5 with the 6^th^ completely unfolded (the native state; N). With increasing temperatures, the unfolded (U) and intermediate states get progressively more stabilized and a Hammond-like movement of the barrier tops is also evident. A closer look at the free-energy profile near the transition mid-point indicates at least two intermediate states as required by chemical-four-state models to reproduce denaturant-dependent kinetics. The major intermediates, I_1_ and I_2_, are centered at values of ∼94 and ∼139 structured residues (Figure 2B) while an early pseudo-intermediate (I′) at ∼74 structured residues also appears as a shoulder to the first barrier.

A simple calculation from the primary sequence boundaries of IκBα indicates that I′, I_1_ and I_2_ correspond to 2, 3 and 4 folded ARs while the primary barrier (following the unfolded state) is located at ∼54 structured residues suggesting that ∼1.5 ARs (i.e. one AR together with the interface with the subsequent AR) have to be folded to nucleate the folding mechanism in line with a previous computational study [24]. For both the intermediates and the primary barrier, it is not clear as to what combination of repeats are involved looking at just the 1D free-energy profile (see below). This analysis is evidence that observing a single sharp transition in experiments is no proof for the lack of intermediates in the folding pathway. The populations of the native and unfolded macrostates (Figure 2C) display a near-sigmoidal transition though the former is significantly broader. The population of the intermediate I_2_ shows a non-monotonic behavior and is maximally populated at 310 K (∼70%) indicating that only ∼4 repeats are fully structured under physiological conditions.

Observation of roll-overs in chevron plots is generally seen as evidence to the presence of intermediates or changes in the rate-limiting step though both are interlinked [41], [42]. As the model naturally predicts the presence of two major intermediates in IκBα, we sought to reproduce the chevron plot by describing the relaxation kinetics as diffusion on the predicted 1D free-energy profile [43]. In experiments, the folding kinetics was monitored from the fluorescence of a sole-tryptophan engineered into the second AR of IκBα (A133W/W258F) [13]. Instead of arbitrarily choosing a signal switch along the free-energy profile we introduce here a simple method to estimate a possible 1D signal for tryptophan fluorescence for this coordinate. We first constructed a minimal 2D structural ensemble, the SSA (Single Sequence Approximation) landscape [19], represented by an ensemble of partially structured states with only a single stretch of folded-like residues. For the 213-residue IκBα this is characterized by 22,791 structured microstates (from N*(N+1)/2, where N is the number of residues in the protein). The interactions between tryptophan and every other residue within a contact cut-off (6 Å) was calculated for each of the microstates (Figure S2) and then projected onto the order parameter thus approximating the tryptophan signal (continuous red line in Figure 2B). The simulated relaxation kinetics using this signal is predicted to be quasi-exponential in the folding limb but bi-exponential at concentrations close to the midpoint in the unfolding limb (3.5–5 M in Figure 2D and Figure S3). The slower phase and the onset of roll-over at ∼5 M urea agree well with experiments despite calibrating the model with purely equilibrium experiments. Bi-exponential kinetics apart from cis-trans proline-isomerization phases has been reported in other AR proteins at precisely the same conditions [23], [44] (i.e. close to the midpoint) highlighting the robustness of the calculation. In an alternate calculation, a simple signal switch was employed at an order parameter value corresponding to the position maximal barrier (54 structured residues; dashed line in Figure 2B). The simulated relaxation kinetics in this scenario is quasi-exponential under most conditions (Figure S3C) mirroring experimental observations while matching with the predictions of the previous calculation (Figure 2D).

**Figure 3:**
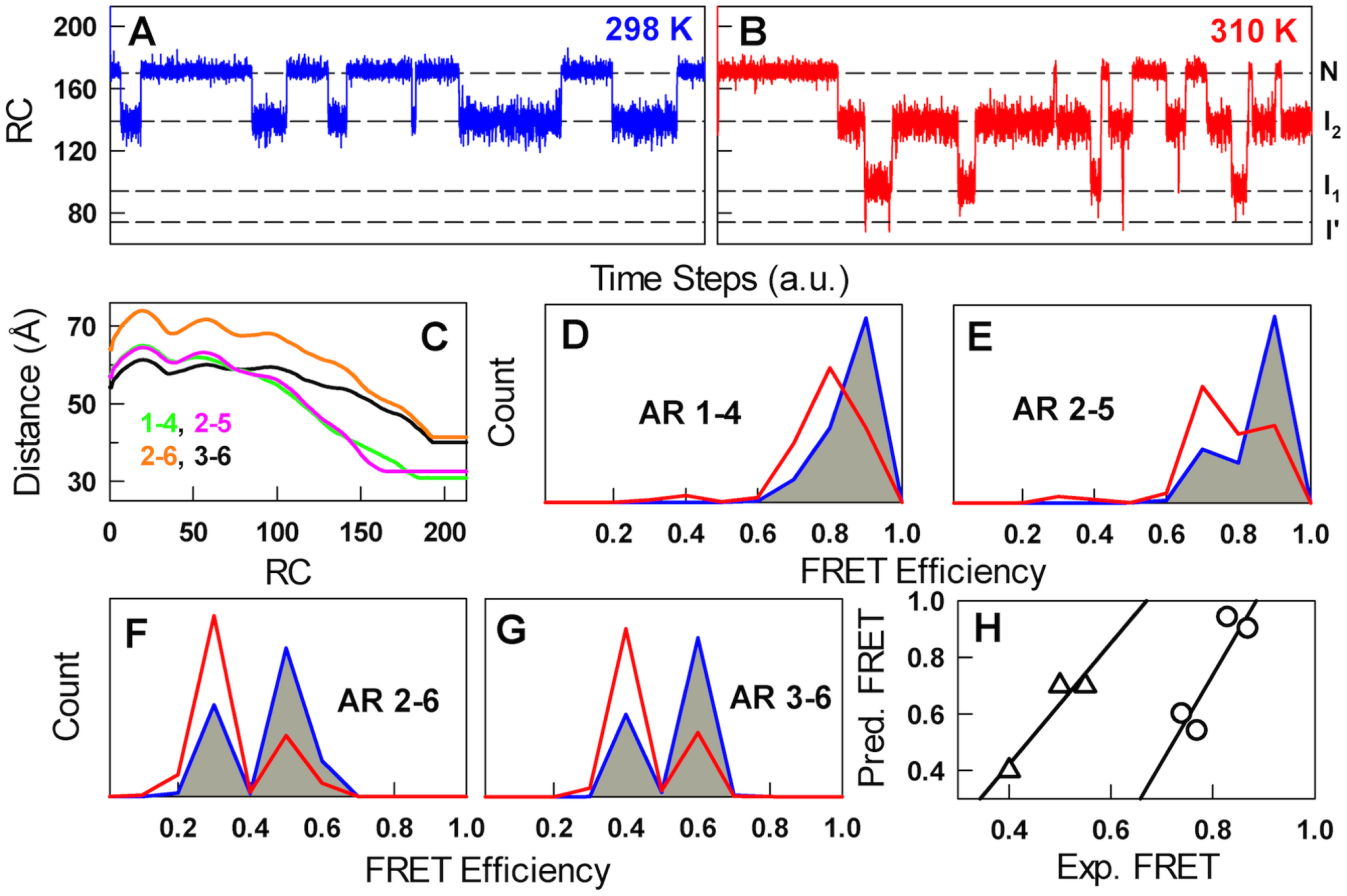
Native state dynamics. (A and B) Single-molecule Monte-Carlo dynamic traces along the reaction coordinate value at two temperatures. (C) Distances between various repeats (1–4, 2–5, 2–6 and 3–6) calculated from the 2D structural ensemble and projected onto the 1D profile as a function of the number of structured residues. (D–G) Binned FRET efficiency histogram indicating that the number of peaks, their positions, and amplitudes are dependent on temperature and on the construct studied. (H) Correlation between experimental and predicted FRET peak positions. Circles correspond to the major peak at 298 K while the triangles indicate FRET position of the minor peaks that appear at 310 K (the data for AR 2–6 is not available).

The agreement between experimental and predicted relaxation kinetics (in both the shape and relative magnitude) suggests that the order parameter –number of structured residues – can also serve as a good reaction coordinate. Can this free-energy profile then be employed to capture and rationalize the smFRET studies on IκBα? [14], [15] Accordingly, we simulated native state dynamics on the 1D free-energy profile by single-bond flip Monte Carlo (MC) dynamics. The simulated MC dynamics reveals an increasingly complex behavior in equilibrium with increasing temperatures (Figure 3A and 3B). At 298 K, only RC values corresponding to 4 (I_2_) and 5 (N) structured repeats are populated while at 310 K the conformational heterogeneity increases to the extent of populating even 3 (I_1_) and occasionally 2 structured repeats (I′) apart from I_2_ and N. To compare against smFRET experiments, distance information is required at the microscopic level, which is generally challenging from the viewpoint of simple models. It should however be much easier for repeat proteins due to the linear nature of their domain organization. To this end, we introduce a novel but simple avenue to reproduce smFRET data by combining the calculated distances between specific residues (as in the experimental construct) for the microstates in the 2D structural ensemble and combining them with distances for the unstructured regions employing the freely-jointed chain model [35] (Figure S4). The distances are then projected onto the RC for each of the construct used in experiments as was done for the estimation of tryptophan fluorescence signal (Figure 3C).

Combining the dynamical information (Figure 3A–B) with the effective distances (Figure 3C) and converting the distances to FRET values using a R_0_ of 51 Å (for the Alexa 555 – Alexa 647 pair) and without invoking any other assumptions we are able to calculate the temperature-dependent FRET dynamics that resemble experiments to a remarkable degree. For example, for donor/acceptor probes located at AR 1 and AR 4 and at 298 K, a broad peak centered at high FRET values is seen (blue in Figure 3D) indicating that the protein is well-folded though with fluctuations from sampling partially-structured conformations (I_2_; Figure 3A). At 310 K, the predicted mean FRET is lower and broader as a result to populating more disordered regions as represented by increasing distances for lower values of RC, exactly as observed in experiments [15]. Though there is a correlation between experiments and simulations on the mean FRET values of the first and second peak (Figure 3H) we do not capture the experimental amplitude, i.e. the relative counts, due to the simplicity of the model and the assumptions involved in calculating the distance along the 1D profile. Despite these shortcomings the semi-quantitative agreement is evidence that the conformational heterogeneity predicted by the model is real and provides a simple and rational explanation to the experimental findings: the dynamics at 310 K can encompass the sampling of as few as three structured repeats (just ∼40% of the protein is folded) and this manifests in the form of changing mean FRET values, increased broadness and extra peaks, relative to the observation at 298 K.

**Figure 4:**
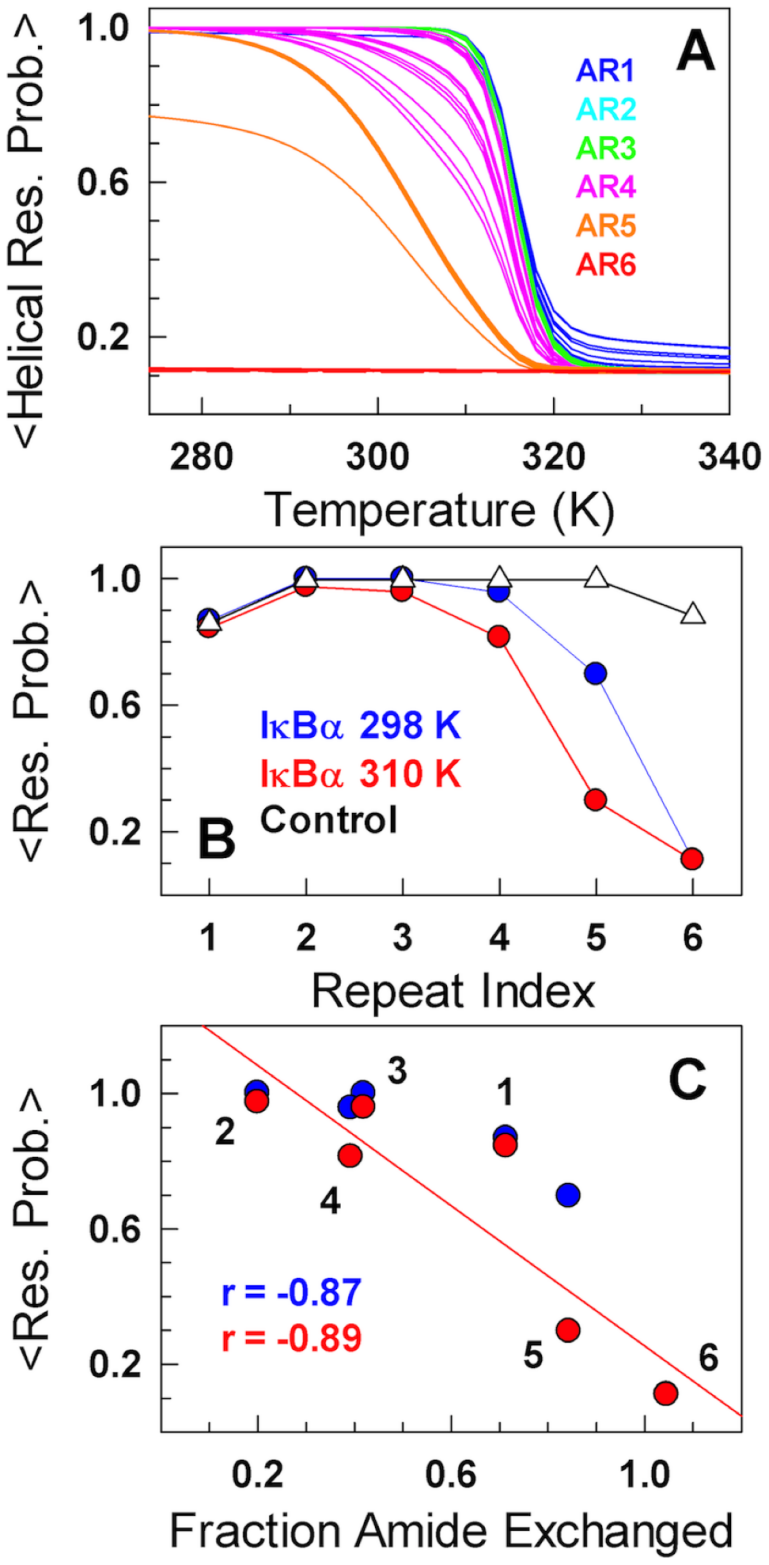
Domino-like destabilization mechanism in the folding of free-IκBα. (A) Global unfolding probabilities of helical residues colored according to the repeat identity. (B) The mean residue probabilities (a larger value indicates higher relative stability) as a function of repeat index at 298 and 310 K. The triangles correspond to the control simulation that employs the contact-map of the bound-IκBα, i.e. employing the entire contact-map from the PDB id. 1NFI without deleting interactions. (C) Correlation between the predicted mean residue unfolding probabilities and the fraction of amides exchanged from experiments at 298 K.

The complex nature of kinetics and smFRET experiments suggests that IκBα should display a correspondingly intricate thermodynamic behavior when monitored at higher detail (residue/atom level) despite displaying two-state-like global thermodynamics. As expected, unfolding curves predicted by the model point to differences in the thermodynamic stabilities of individual repeats evident in the form of variable transition mid-points (Figure 4A). We find that AR6 is completely unfolded under all conditions while AR5 undergoes a non-cooperative transition from ∼80% folded at 298 K to just ∼35% folded under physiological conditions (310 K). AR4 is also partially destabilized due to minimal interactions with AR5 (Figure 4A) while the repeats 1–3 are reasonably well structured at 298 and 310 K. This suggests that the partial of unfolding of AR repeats 4 and 5 primarily contribute to the steep pre-transition observed in equilibrium experiments, An alternate procedure to visualize the difference in stability is to plot the mean residue probability (a proxy to stability without a recourse to two-state model fitting) as a function of repeat index (Figure 4B). This clearly reveals the difference in stability between repeats and their temperature dependence. It also shows that AR 1 is less stable than ARs 2 and 3 due to end-effects as one of its interfaces is completely exposed to the solvent in contrast to the central repeats, while ARs 5 and 6 are significantly unfolded in agreement with experiments [37]. The thermodynamic manifestation of this observation should be captured in hydrogen/deuterium exchange (HX) experiments that can report on the degree of exchange of amide protons arising from structural fluctuations. In fact, HX experiments at 298 K reveal the following order of protection - AR 2>3,4>1>5>6 [11]- and is highly correlated with the predicted mean residue probabilities of individual repeats at both 298 and 310 K (Figure 4C).

## Discussion

Repeat proteins are generally symmetric both structurally and at the level of sequences. At the structural level, every repeat has two inter-repeat interfaces while the end-repeats (at the N- and C-terminii) are characterized by one interface. At the sequence level, there is a high degree of similarity that enables the repeats to adopt the ankyrin fold. This has been exploited to engineer super-stable AR proteins simply by tuning the agreement with consensus sequences [37], [45]–[48]. What happens when this symmetry is broken or when one of the terminal repeats is ‘designed’ to be less structured than the rest? In other words, what is the origin, at the sequence level, of this unique conformational behavior in IκBα? Sequence alignment and mutational studies indicate that AR 6 lacks a particular consensus motif (TPLHLA) that makes it less stable and prone to disorder [12]. We find that this evolutionarily selected lack of structure in AR 6 translates into a loss of one inter-repeat interface for AR 5. This in turn destabilizes AR 5 and hence AR 4 to a lesser extent (as shown in Figure 4B) highlighting the critical nature of inter-repeat interactions and is in agreement with expectations based on analytical and coarse-grained models [24], [49]. In other words, we identify a novel domino-like effect of disordered regions in IκBα involving successive destabilization of nearby repeats that might be functionally important (see below) arising from simple symmetry-breaking design by Nature. A control simulation with ARs 5 and 6 fully folded reveals only marginal destabilization of repeats and is seen only in ARs 1 and 6 due to the lack of one contact interface, i.e. the end effects (also seen in AR 1 of IκBα; Figure 4B). From a thermodynamic perspective, the disorder induced domino-like destabilization mechanism drives the dynamics under native conditions with ARs 2 and 3 displaying minimal equilibrium fluctuations relative to other repeats (Figure 4). Accordingly, from the viewpoint of folding, the AR 2 or 3 should fold first, followed by ARs 4, 1, 5 and 6 in that order (on average) as inferred from HX experiments akin to the nucleation-condensation mechanism. It is, however, important to note that AR 5 is not fully folded even at 298 K while AR 6 is completely unstructured under all conditions.

**Figure 5:**
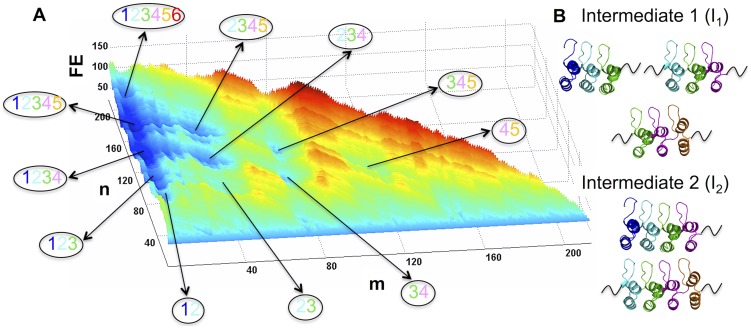
Folding landscape and complexity. (A) SSA free-energy landscape of ligand-free IκBα highlighting the diversity of intermediates (lower free energy – FE - and blue in color) that can be populated during folding. The coordinates (*m, n*) represent the starting residue and the number of structured residues, respectively. The fully folded state is therefore (1, 213), i.e. starting from 1 there are 213 folded residues, while a partially structured state with ARs 2, 3, and 4 folded will be centered around (35, 90), i.e. starting from 35 there are 90 structured residues. The partially folded repeats in each of the local minima are indicated as numbers within ellipses following the color code of Figure 1A. (B) Structural view of the intermediates I_1_ and I_2_. The black curves indicate unstructured regions.

The offshoot of the disorder-induced domino effect is an intricate conformational landscape characterized by several valleys and ridges and hence numerous metastable states that can be accessible by equilibrium fluctuations (Figure 5A and 3A–B). The valleys or local minima in free-energy (dark blue in Figure 5A) along the SSA landscape correspond to the intermediate states as also seen in the 1D free-energy profile (Figure 2A and 2B). Since there is a direct structural interpretation, we map the corresponding minima to specific partially structured states as shown in Figure 5B and 5C. The intermediate state 1 (I_1_) corresponds to structure in three repeats (ARs 123, 234, 345 in that order of decreasing probability) while I_2_ involves contributions from ARs 1234 and 2345. This near-continuous residue-level SSA landscape is very similar to the experimentally constructed discrete (at the repeat level) landscape of the notch AR protein [50] but with the associated barriers apart from the intermediates. Eliminating the equilibrium pre-transition by assuming ARs 5 and 6 to be fully folded (i.e. similar to the crystal structure) dramatically alters the landscape that becomes much simpler with the population of very few intermediate species (control in Figure 4B Figure S5B). Experimentally, the half-life of mutant IκBα with fully structured ARs 5 and 6 is increased to 23 minutes (compared to 7 minutes for the wild-type) while remarkably binding weaker to NF-κB thus affecting the NF-κB signaling module *in vivo* [12]. This is indirect evidence that disorder in repeat 6 and the related landscape and dynamics that we extract here might be required for proper functioning of IκBα.

In effect, our theoretical analysis of the IκBα folding behavior provides a structural view of the populating intermediates and explains the diverse and sometimes conflicting dynamic and thermodynamic experimental observations. We have, in parallel, devised a methodology to model disorder in systems that fold upon binding by modifying the contact map guided by equilibrium experimental observables and model smFRET experimental data in linear systems in combination with elementary polymer physics treatments. The resulting conformational landscape of IκBα is predicted to be complex with multiple unstructured states populated even in equilibrium. This directly explains the short half-life of IκBα inside cells as proteins with unstructured regions (in this case arising from intermediates or metastable states) are targeted for degradation thus temporally regulating their biological activity [51]. The inability of the simple kinetic model to capture the roll-over in the folding arm of the chevron and over-prediction of relaxation rates on either side of the denaturation midpoint is evidence that a single diffusion coefficient might not be sufficient to explain the dynamics due to either the changing nature of the equilibrium ensemble with conditions (as shown in the one-state downhill folder BBL [52]) or due to the effect of parallel/competing pathways and stable compact intermediates [48], [53]–[55].

Promiscuous binding in single domain proteins can be possibly achieved by downhill-folding systems that respond to stimuli/conditions by changing the dimensions of the ensemble through gradual unfolding [29], [56], [57] (the ‘molecular rheostat’ hypothesis [29]). An ensemble approach to multi-domain systems suggests that the degree and magnitude of coupling between individual domains can result in a variety of functional behaviors [58]. Analytical models of repeat proteins further predict a delicate balance between intra- and inter-repeat interactions that enable them to sample functionally relevant conformations [24]. We find a similar feature in IκBα wherein the dimensions, nature and dynamics of the ensemble are determined by a disorder-induced domino effect that can selectively tune the degree of unfoldedness of individual repeats. This, we propose, is critical for its promiscuous binding as a heterogeneous native ensemble under physiological conditions can enable IκBα to bind several different partners and possibly explains the large differences in binding constants of characterized IκBα protein pairs (*K_d_* ∼40 pM –217 nM) [4], [9]. The folded ARs are generally seen as scaffolds on to which the more functionally relevant and weakly structured ARs are linked [59]. The disorder induced destabilization mechanism of repeats we propose goes beyond the mere scaffold functionality and points to a simple strategy employed by Nature to load multiple functionalities into repeat proteins while at the same time imposing a tight temporal control through disorder.

## Supporting Information

Figure S1 **Differential scanning calorimetry and chemical denaturation.** (A) Experimental excess heat capacity profile of IκBα (blue circles) together with the fit from the WSME model (red). A highly sloped pre-transition is also observed in the DSC simulation, which is absorbed by the native baseline. This manifests in the excess heat capacity in the form of a gradual low temperature dependence. No exact fits were performed as aggregation artifacts have been reported in the experimental work. (B) Predicted chemical unfolding curve at 278 K. In this case, all parameters are fixed to that from thermal denaturation while the *m^cont^* is tuned to reproduce the experimental midpoint denaturant concentration of 3.6 M urea. The resulting *m^cont^* value is −0.12 J mol^−1^ M^−1^ per native contact that translates to a global *m*-value of 7.3 kJ mol^−1^ M^−1^, which is similar to the experimental numbers (∼7.5–7.9 kJ mol^−1^ M^−1^).
(TIF)

Figure S2 **Estimation of tryptophan signal from the SSA structural ensemble.** The number of contacts formed by tryptophan (of the A133W mutant) represented in the minimal 2D structural ensemble: m – the starting residue, n – number of structured residues. This is similar to the SSA free-energy landscape but instead of free energy the number of contacts are plotted.
(TIF)

Figure S3 **Folding kinetics of IκBα.** The kinetic decays as monitored by a tryptophan signal were simulated by mimicking the experimental protocol wherein re-folding (0–3.4 M urea) is initiated from an unfolded ensemble at 5 M urea and unfolding (3.6–9 M urea) is initiated from the native ensemble at 0 M urea. (A & B) Relaxation decays and double-exponential fits (panel A; blue and red, respectively) together with the fast- and slow-phase amplitudes (panel B) for the projected signal calculated form the 2D structural ensemble. (C) Relaxation decays together with the single-exponential fits to the single phase from the tryptophan signal represented by a signal switch at an order parameter value of 54. At high urea concentrations (>6 M), we obtain compressed exponentials due to the instantaneous nature of the signal switch, compared to only minimal deviations from single-exponential decays in panel A at 7 M.
(TIF)

Figure S4 **Estimation of end-to-end distances from the SSA structural ensemble.** Panels A-D show a plot of the expected distance between the FRET donor/acceptor probes located at AR 1–4, 2–5, 2–6, and 3–6 for the 2D structural ensemble. The scale to the right of panel A indicates the coloring code in Å and is maintained through all panels.
(TIF)

Figure S5 **Effect of disorder on the folding landscape of IκBα.** Panel A plots the SSA free energy landscapes at 310 K for the free IκBα with the modified contact map (upper left triangle of Figure 1B in the main text) wherein contacts are systematically eliminated to match the experimental pre-transition amplitude, i.e. to mimic the lack of structure in ARs 5 and 6. Panel B plots the SSA landscape calculated without modifying the contact map (lower right triangle of Figure 1B in the main text), i.e. with fully folded ARs 5 and 6 (for example, Bcl-3). The scales to the right of the panels denote the identical coloring code of free energy in units of kJ mol^−1^. It is clear by comparison that the lack of structure in ARs 5 and 6 dramatically alters the folding landscape by reducing the barriers thus enabling almost the entire landscape accessible through conformational fluctuations.
(TIF)

Text S1 **Details of the WSME model and the parameters.**
(PDF)
