# Supplementary figures and images for: A Disorder-Induced Domino-Like Destabilization Mechanism Governs the Folding and Functional Dynamics of the Repeat Protein IκBα
Source: PLoS Comput Biol. 2013 Dec 19;9(12):e1003403. doi: 10.1371/journal.pcbi.1003403 (PMC3868533; doi:10.1371/journal.pcbi.1003403)

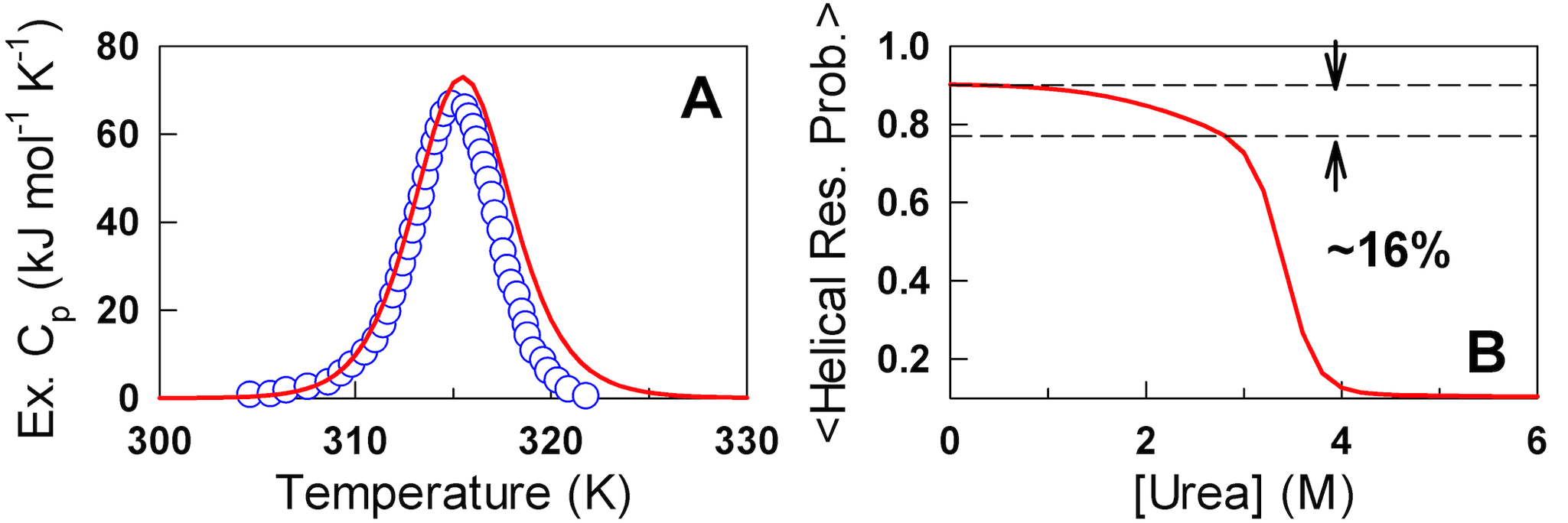

Supplement: Figure S1 — Differential scanning calorimetry and chemical denaturation. (A) Experimental excess heat capacity profile of IκBα (blue circles) together with the fit from the WSME model (red). A highly sloped pre-transition is also observed in the DSC simulation, which is absorbed by the native baseline. This manifests in the excess heat capacity in the form of a gradual low temperature dependence. No exact fits were performed as aggregation artifacts have been reported in the experimental work. (B) Predicted chemical unfolding curve at 278 K. In this case, all parameters are fixed to that from thermal denaturation while the mcont is tuned to reproduce the experimental midpoint denaturant concentration of 3.6 M urea. The resulting mcont value is −0.12 J mol−1 M−1 per native contact that translates to a global m-value of 7.3 kJ mol−1 M−1, which is similar to the experimental numbers (∼7.5–7.9 kJ mol−1 M−1). (TIF) [file pcbi.1003403.s001.tif]

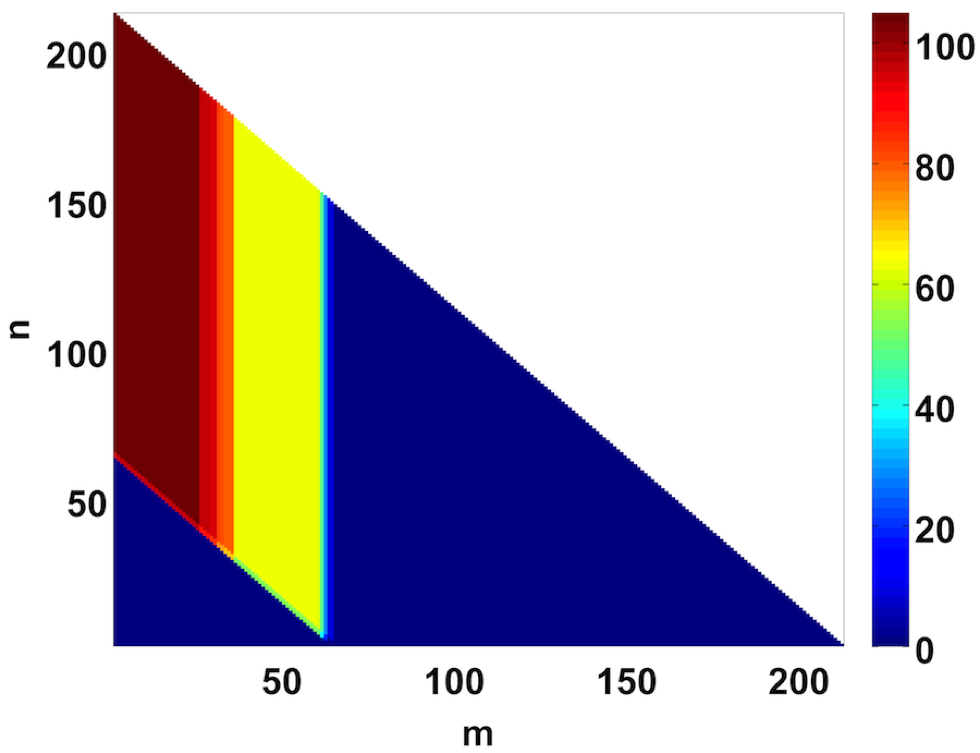

Supplement: Figure S2 — Estimation of tryptophan signal from the SSA structural ensemble. The number of contacts formed by tryptophan (of the A133W mutant) represented in the minimal 2D structural ensemble: m – the starting residue, n – number of structured residues. This is similar to the SSA free-energy landscape but instead of free energy the number of contacts are plotted. (TIF) [file pcbi.1003403.s002.tif]

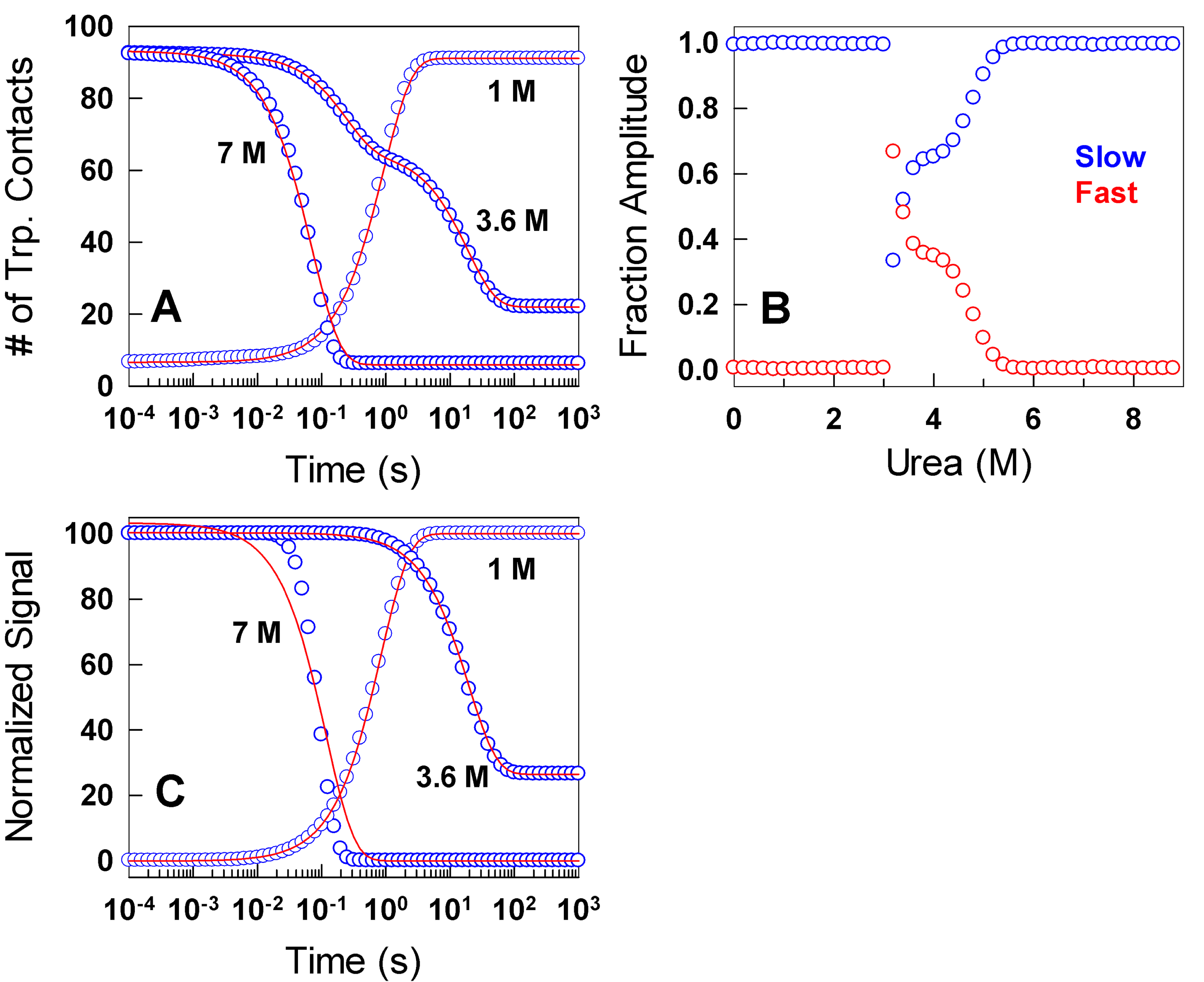

Supplement: Figure S3 — Folding kinetics of IκBα. The kinetic decays as monitored by a tryptophan signal were simulated by mimicking the experimental protocol wherein re-folding (0–3.4 M urea) is initiated from an unfolded ensemble at 5 M urea and unfolding (3.6–9 M urea) is initiated from the native ensemble at 0 M urea. (A & B) Relaxation decays and double-exponential fits (panel A; blue and red, respectively) together with the fast- and slow-phase amplitudes (panel B) for the projected signal calculated form the 2D structural ensemble. (C) Relaxation decays together with the single-exponential fits to the single phase from the tryptophan signal represented by a signal switch at an order parameter value of 54. At high urea concentrations (>6 M), we obtain compressed exponentials due to the instantaneous nature of the signal switch, compared to only minimal deviations from single-exponential decays in panel A at 7 M. (TIF) [file pcbi.1003403.s003.tif]

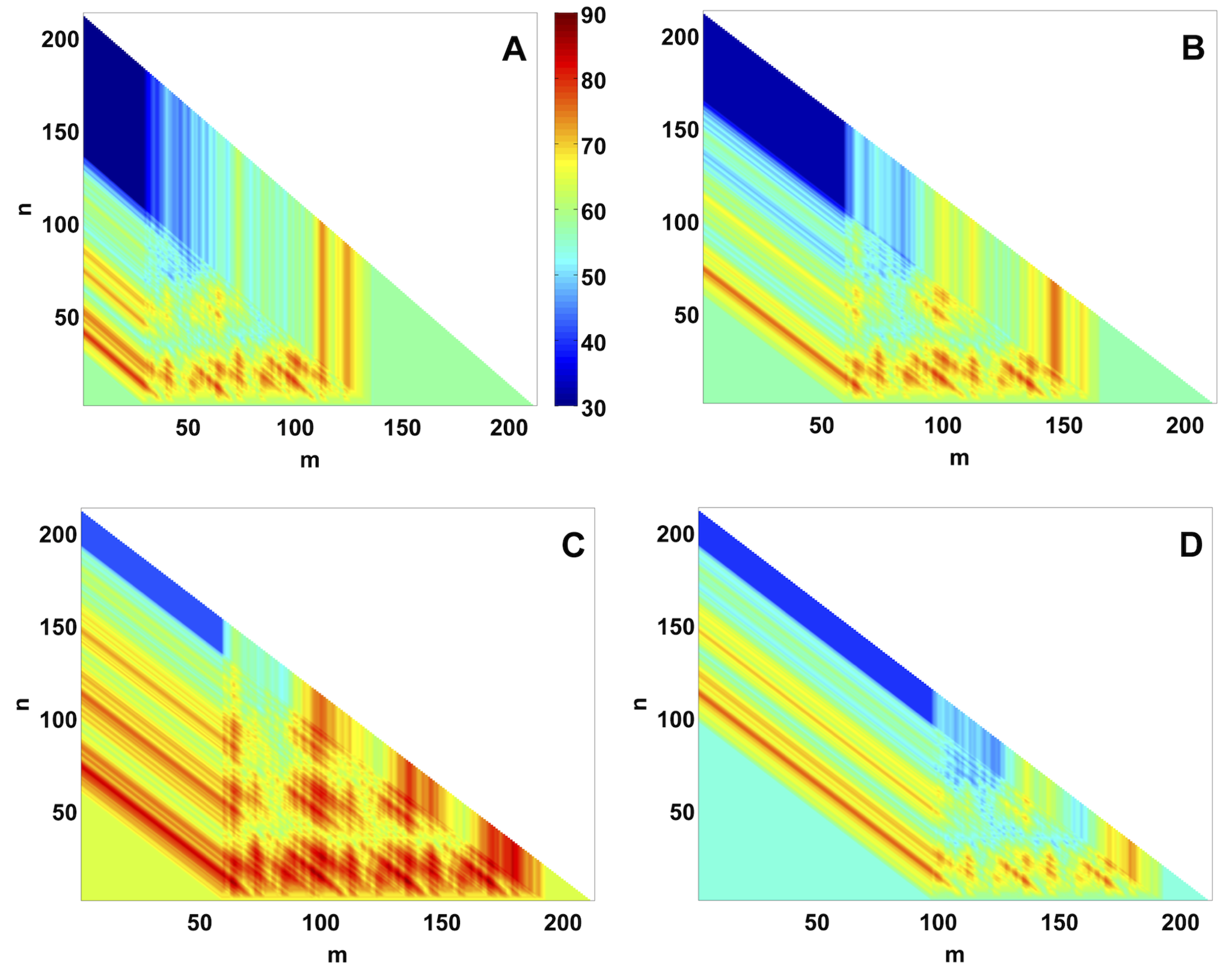

Supplement: Figure S4 — Estimation of end-to-end distances from the SSA structural ensemble. Panels A-D show a plot of the expected distance between the FRET donor/acceptor probes located at AR 1–4, 2–5, 2–6, and 3–6 for the 2D structural ensemble. The scale to the right of panel A indicates the coloring code in Å and is maintained through all panels. (TIF) [file pcbi.1003403.s004.tif]

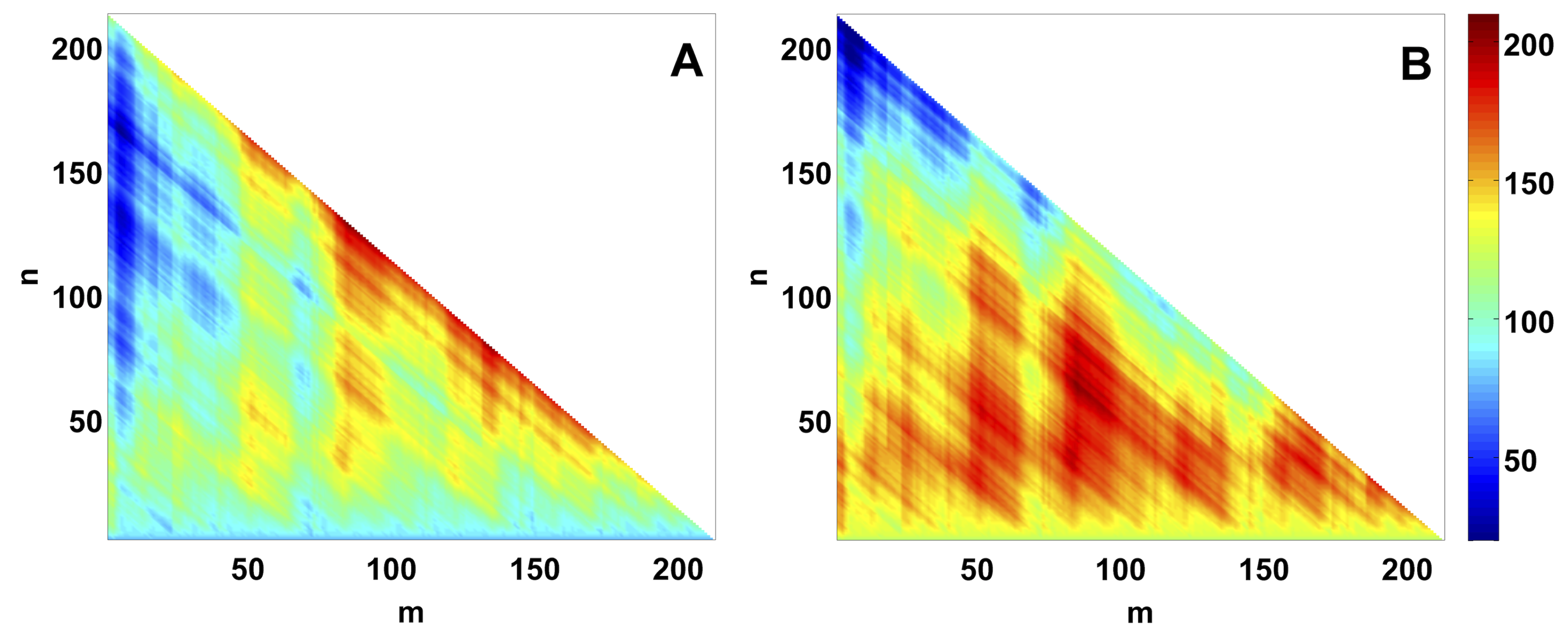

Supplement: Figure S5 — Effect of disorder on the folding landscape of IκBα. Panel A plots the SSA free energy landscapes at 310 K for the free IκBα with the modified contact map (upper left triangle of Figure 1B in the main text) wherein contacts are systematically eliminated to match the experimental pre-transition amplitude, i.e. to mimic the lack of structure in ARs 5 and 6. Panel B plots the SSA landscape calculated without modifying the contact map (lower right triangle of Figure 1B in the main text), i.e. with fully folded ARs 5 and 6 (for example, Bcl-3). The scales to the right of the panels denote the identical coloring code of free energy in units of kJ mol−1. It is clear by comparison that the lack of structure in ARs 5 and 6 dramatically alters the folding landscape by reducing the barriers thus enabling almost the entire landscape accessible through conformational fluctuations. (TIF) [file pcbi.1003403.s005.tif]
