# Supplementary material for: A Disorder-Induced Domino-Like Destabilization Mechanism Governs the Folding and Functional Dynamics of the Repeat Protein IκBα
Source: PLoS Comput Biol. 2013 Dec 19;9(12):e1003403. doi: 10.1371/journal.pcbi.1003403 (PMC3868533; doi:10.1371/journal.pcbi.1003403)
Supplement: Text S1 — Details of the WSME model and the parameters. (PDF) [file pcbi.1003403.s006.pdf]

Supporting Text S1

A Disorder Induced Domino-Like Destabilization Mechanism Governs the Folding and Functional Dynamics of the Repeat Protein IκBα

Srinivasan Sivanandan[1] & Athi N. Naganathan[2]*

[1]Department of Biotechnology, Indian Institute of Technology Kharagpur, Kharagpur 721302, India.
[2]Department of Biotechnology, Indian Institute of Technology Madras, Chennai 600036, India.

The Wako-Saitô-Muñoz-Eaton (WSME) model

The free-energy of a particular microstate (*m,n*) (i.e., a string of 1's between and including *m* and *n*) in the WSME model with electrostatics and solvation free-energy [19,21] is given by

$$\Delta F = \sum \Delta G^{stab}_{m,n} - T\sum_{m}^{n} \Delta S_{conf} \tag{S.1}$$

The stabilization free-energy of a microstate (first term in the above equation) is represented as a sum of van der Waals interactions (E_{VdW}), electrostatic potential (E_{elec}) and solvation free-energy (ΔG_{solv}) arising from purely native contributions:

$$\Delta G^{stab}_{m,n} = E_{VdW} + E_{elec} + \Delta G_{solv} \tag{S.2}$$

The second term corresponds to the entropic cost of fixing residues to the native conformation at the temperature *T*. The interacting partners that contribute to the van der Waals energy are identified by setting a distance cut-off (r_{cut}) to the pair-wise heavy-atom partners (*i, j*) calculated from the PDB file.

$$E_{VdW} = \sum_{m,n} \xi_{i,j}\rho \tag{S.3}$$

where $\rho = 1$ if $r_{ij} \le r_{cut}$ and $\rho = 0$ otherwise. In atomic-level force-fields the interaction energies are distance dependent but since the WSME model pre-assumes an ensemble derived from the native structure a distance dependent energy is not required. The r_{cut} was fixed to 6 Å and nearest neighbors were excluded from the contact calculation as before[19,21].

A Debye-Hückel (DH) treatment is employed for the electrostatic potential term:

$$E_{elec} = \sum_{m,n} K_{Coulomb} \frac{q_i q_j}{\varepsilon_{eff} r_{ij}} \exp\left(-r_{ij}\kappa\right) \tag{S.4}$$

wherein $K_{Coulomb}$ is the Coulomb constant (1389 kJ.Å/mol), q_i is the charge on the atom i, r_{ij} is the distance between charge centers i and j, and ε_{eff} is the effective dielectric constant and is fixed to 29. $1/\kappa$ is the Debye screening length that depends on ε_{eff}, solvent ionic-strength (I) and temperature (T) as

$$\kappa^2 = \frac{8\pi e^2 I}{\varepsilon_{eff} k_B T} \tag{S.5}$$

where k_B is the Boltzmann's constant, and e is the elementary charge. Charges were assigned to atoms according to pH 7.0 protonation state and I was fixed to the experimental value of 0.05 M[10].

The solvation free-energy is approximated as being proportional to the number of formed contacts ($x_{cont}^{m,n}$) in that microstate with the proportionality constant being ΔC_p^{cont}, which is the temperature-independent heat capacity change upon fixing a native contact. Therefore,

$$\Delta G_{solv} = x_{cont}^{m,n} \Delta C_p^{cont} \left[\left(T - T_{ref} \right) - T \ln\left(T / T_{ref} \right) \right] \tag{S.6}$$

where T_{ref} is the reference temperature which is fixed to 385 K[34]. A uniform conformational entropy cost (ΔS_{conf}) is assigned to residues independent of sequence or native secondary-structure.

The partition function (Z) is calculated employing the transfer-matrix formalism of Wako and Saitô[16,17]:

$$Z\left(T\right) = v_l \left[\prod_{i=1}^{N} X_i \right] v_r^{tr} \tag{S.7}$$

where

$$X_i = \begin{pmatrix} 1 & 1 & 1 & \cdots & 1 & 1 \\ z & 0 & 0 & \cdots & & 0 \\ 0 & H_1^{(i)} z & 0 & & & \vdots \\ & & H_2^{(i)} z & & 0 & 0 \\ & \mathbf{0} & & & H_{N-2}^{(i)} z & H_{N-1}^{(i)} z \end{pmatrix}$$

$$v_l = \left(1,1,1,\cdots,1\right)$$

$$v_r = \left(1,0,0,\cdots,0\right)$$

and

$$H_k^{(i)} = \exp\left(-\beta \sum_{j=1}^{k} \Delta G^{stab} \right) \qquad (k \le N-i) \tag{S.8}$$

$$H_k^{(i)} = 0 \qquad (k > N-i)$$

Here, $\beta = 1/RT$ and $z = \exp\left(\Delta S_{conf} / R\right)$ where R is 8.314 J mol^{-1} K^{-1}. The one-dimensional free-energy profiles are constructed by accumulating the total statistical weight for members of the ensemble with different number of structured residues.

The overall probability of a particular residue to be folded (p_f^i) can be obtained from

$$p_f^i = Z^{-1} v_l \left[\prod_{j=1}^{i-1} X_j \right] \left[\frac{\partial X_i}{\partial \ln z} \right] \left[\prod_{j=i+1}^{N} X_j \right] v_r^{tr} \tag{S.9}$$

The final parameters from fit to the excess heat capacity profile are: ΔS_{conf} = -18.1 J mol^{-1} K^{-1}, ε = -70.1 J mol^{-1} while ΔC_p^{cont} is fixed to -0.358 J mol^{-1} K^{-1}[19].

Chemical Denaturation

Chemical denaturation effects are introduced by a phenomenological constant that decreases the stabilization free-energy linearly with denaturant concentration ([D]):

$$\Delta G_{m,n}^{stab}\left([D]\right) = E_{VdW} + E_{elec} + \Delta G_{solv} - x_{cont}^{m,n} m^{cont} [D] \tag{S.10}$$

Other parameters including entropic cost were fixed to the original values extracted from the characterization of temperature-induced denaturation.
